# Cancer Associated Fibroblasts and Senescent Thyroid Cells in the Invasive Front of Thyroid Carcinoma

**DOI:** 10.3390/cancers12010112

**Published:** 2020-01-01

**Authors:** Emanuela Minna, Silvia Brich, Katia Todoerti, Silvana Pilotti, Paola Collini, Elisa Bonaldi, Paola Romeo, Loris De Cecco, Matteo Dugo, Federica Perrone, Adele Busico, Andrea Vingiani, Ilaria Bersani, Andrea Anichini, Roberta Mortarini, Antonino Neri, Giancarlo Pruneri, Angela Greco, Maria Grazia Borrello

**Affiliations:** 1 Molecular Mechanisms Unit, Department of Research, Fondazione IRCCS Istituto Nazionale dei Tumori, 20133 Milan, Italy; 2 Laboratory of Molecular Pathology, Department of Pathology, Fondazione IRCCS Istituto Nazionale dei Tumori, 20133 Milan, Italy; 3 Hematology, Fondazione Cà Granda IRCCS Policlinico, 20122 Milan, Italy; 4 Department of Pathology, Fondazione IRCCS Istituto Nazionale dei Tumori, 20133 Milan, Italy; 5 Soft Tissue and Bone Pathology, Histopathology and Pediatric Pathology Unit, Department of Diagnostic Pathology and Laboratory Medicine, Fondazione IRCCS Istituto Nazionale dei Tumori, 20133 Milan, Italy; 6 Platform of Integrated Biology, Department of Applied Research and Technology Development, Fondazione IRCCS Istituto Nazionale dei Tumori, 20133 Milan, Italy; 7 School of Medicine, Università degli Studi di Milano, 20122 Milan, Italy; 8 Human Tumors Immunobiology Unit, Department of Research, Fondazione IRCCS Istituto Nazionale dei Tumori, 20133 Milan, Italy; 9 Department of Oncology and Hemato-oncology, University of Milan, 20122 Milan, Italy

**Keywords:** thyroid cancer, CAFs, senescent cells, BRAFV600E, BRAF- and RAS-like signaling

## Abstract

Thyroid carcinoma (TC) comprises several histotypes with different aggressiveness, from well (papillary carcinoma, PTC) to less differentiated forms (poorly differentiated and anaplastic thyroid carcinoma, PDTC and ATC, respectively). Previous reports have suggested a functional role for cancer-associated fibroblasts (CAFs) or senescent TC cells in the progression of PTC. In this study, we investigated the presence of CAFs and senescent cells in proprietary human TCs including PTC, PDTC, and ATC. Screening for the driving lesions *BRAFV600E* and *N/H/KRAS* mutations, and gene fusions was also performed to correlate results with tumor genotype. In samples with unidentified drivers, transcriptomic profiles were used to establish a BRAF- or RAS-like molecular subtype based on a gene signature derived from The Cancer Genome Atlas. By using immunohistochemistry, we found co-occurrence of stromal CAFs and senescent TC cells at the tumor invasive front, where deposition of collagen (COL1A1) and expression of lysyl oxidase (LOX) enzyme were also detected, in association with features of local invasion. Concurrent high expression of CAFs and of the senescent TC cells markers, *COL1A1* and *LOX* was confirmed in different TC histotypes in proprietary and public gene sets derived from Gene Expression Omnibus (GEO) repository, and especially in *BRAF* mutated or BRAF-like tumors. In this study, we show that CAFs and senescent TC cells co-occur in various histotypes of BRAF-driven thyroid tumors and localize at the tumor invasive front.

## 1. Introduction

The vast majority (98%) of thyroid tumors arise from thyroid follicular epithelial cells and comprise various histological types and variants, ranging from well differentiated thyroid carcinomas (WDTCs) to poorly differentiated (PDTCs) and undifferentiated (anaplastic, ATCs) forms. WDTCs account for most of follicular cell derived TCs (80–90%) and comprise papillary and follicular thyroid carcinoma (PTC and FTC, respectively) among which PTC (85%) definitively outnumbers FTC (15%). PDTC and ATC are rare tumors that can arise de novo from follicular cells or develop from pre-existing PTCs or FTCs [1] according to a model of sequential progression from low-grade to high-grade thyroid tumors.

Although they are annotated as well differentiated forms, PTC and FTC differ in terms of nuclear features, growth patterns, and the route for metastases dissemination. PTC displays papillary or follicular growth patterns and metastatic spreading preferentially via lymphatic vessels, while FTC displays follicular growth pattern and dissemination preferentially via blood vessels [2].

In addition to the histopathological classification, studies aimed at the identification of molecular events driving TC have recently provided evidence that specific genetic alterations play a role in the carcinogenesis of various thyroid tumors [1]. Most of the identified driving lesions involve the effectors of MAPK and PI3K-AKT signaling pathways. *BRAF* mutations (mainly *BRAFV600E*) as well as *RET* and *TRK* gene fusions are more frequently detected in PTC, while *RAS* mutations are more frequent in FTC [3]. An exception is represented by the follicular variant of PTC that shares histological features with both PTC and FTC and harbors frequent *RAS* mutations [4]. Gene lesions associated with well-differentiated forms, in particular *BRAF* and *RAS* mutations, are also frequently found in PDTC and ATC in association with additional alterations (such as *PI3K*, *PTEN*, *TP53* and *TERT* promoter mutations [5]), supporting the model of tumor progression from pre-existing PTC or FTC driven by sequential accumulation of multiple genetic abnormalities.

Along with the major drivers, however, less frequent and/or unconventional genetic alterations were identified in TCs, but the molecular effects remain to be clarified. To achieve this aim, a major improvement has been made by a remarkable study from The Cancer Genome Atlas (TCGA), which investigated a large PTC cohort by using a comprehensive multi-omics approach [6]. The study not only expanded the genomic landscape of PTC, but also proposed an improved molecular classification of PTC based on gene expression profiles that describe tumor properties better than pathological classifications [6]. The authors established a 71-gene signature, indicative of MAPK pathway transcriptional activation, that permits to classify *BRAFV600E*- and *RAS*-mutated PTCs and to determine whether a given tumor, characterized by other less common or unknown alterations, resembles one of these two molecular subtypes, defining a BRAF-like or RAS-like signaling type, respectively. The TCGA-based BRAF- or RAS-like signaling classification was then confirmed in subsequent studies not only in additional cohorts of PTC [7,8], FTC [8], PDTC, and ATC [5], but also in case lists including both tumoral and non tumoral thyroid tissues (reference [7] and our unpublished data).

While collectively the definition of gene lesions and expression profiles have greatly expanded our understanding of the genetic basis of TC, the way cancer cells interact with the tumor microenvironment (TME) still represents a poorly investigated field in the context of thyroid cancer.

Several lines of evidence have shown how tumor growth and progression can be affected by cellular (both of immune and not immune origin) and TME-related components [9]. Many genes encoding secreted factors with paracrine effects on TME components such as, for instance, stromal cells (e.g., fibroblasts) and/or extracellular matrix proteins (e.g., collagen), have been found to be abnormally expressed in tumor tissues [9].

Recently Jolly et al. have described a mouse model driven by the thyroid-specific expression of oncogenic *BRAFV600E* where the converging action of thyroid tumor cells and stromal cancer-associated fibroblasts (CAFs) promote TC progression [10]. According to the model, CAFs are recruited in the stroma at the tumor invasive front where they synthesize and deposit collagen (COL1A1), which is in turn cross-linked by the enzyme LOX, produced by thyroid tumor cells. Collectively, this coordinated action leads to matrix stiffness and progression from PTC to less differentiated PDTC. Interestingly, the authors reported this feature specifically in murine thyroid tumors driven by *BRAFV600E* mutation, but not in those driven by *HRAS* mutation. As validation of the proposed model, they investigated the expression of *LOX* and *COL1A1* genes in human TC by using two public datasets [6,11] and confirmed their upregulation in thyroid tumors as well as the association with *BRAF* mutation, but not *RAS* mutation. The concurrent involvement and expression of CAF markers in human TC, however, remained unexplored.

Another contribution in understanding the process of thyroid tumor progression, specifically in *BRAFV600E* mutated PTCs, has been recently provided by Kim et al. who reported the active involvement of senescent thyroid cancer cells (senescent TC cells) in the invasion and metastases of PTC [12]. Cellular senescence is a feature of steady status cell cycle arrest that can be induced by a variety of stress stimuli, including oncogene activation [12]. In this specific case, it is defined as oncogene induced senescence (OIS). The occurrence of OIS in thyroid has been already described in reference [13], as well as by our laboratory [14,15]. Along with cell cycle arrest, confirmed by increased expression of cell cycle inhibitors as p16^INK4a^ and p21^CIP1^, another feature of senescent cells is to be metabolically active, secreting a myriad of growth factors, cytokines, and chemokines collectively termed SASP (senescence-associated secretory phenotype). The nature and composition of SASP varies depending on cellular context and can display pro- or anti-tumoral properties [16]. Kim et al. starting from the observation that senescent thyroid cells were frequently detected at the invasive front of human *BRAFV600E*-expressing PTCs, determined through subsequent in vitro and in vivo studies that these cells constitute a subpopulation of the thyroid tumoral cells and secrete factors that are able to stimulate the collective migration and invasion of the surrounding TC cells, thus promoting tumor invasiveness. Whether senescent TC cells secretome was able to affect additional players in TME was not explored.

In this study, we exploited a retrospective case list of human thyroid tissues collected at our Institution, and included various tumor histotypes as well as paired and non-paired non-neoplatic thyroid (NT) controls, to investigate through immunohistochemical (IHC) and gene expression analyses whether the cross-talk between thyroid tumor cells and CAFs described in Jolly et al.’s mouse model also occurs in human TC, and whether senescent thyroid cells may be potentially involved in this process.

In addition, we exploited tumor genetic characterization, both at molecular and transcriptomic levels, to correlate the results with the tumor genotype. An additional cohort of 407 human thyroid tissues was derived from public gene expression studies and tested as a validation set.

## 2. Results

### 2.1. CAFs Are Enriched at the Invasive Front of Human Thyroid Cancers

The presence of CAFs was investigated by using immunostaining with alpha-smooth muscle actin (α-SMA) in a series of 65 formalin-fixed paraffin-embedded (FFPE) tissue sections including thyroid tumors (of different histotypes, details are provided in Table S1) and NTs derived from 48 patients (43 TC patients and 5 NT controls). For 32 FFPE tumor sections with available adjacent NT, the paired non tumoral thyroid was also scored.

**Figure 1:**
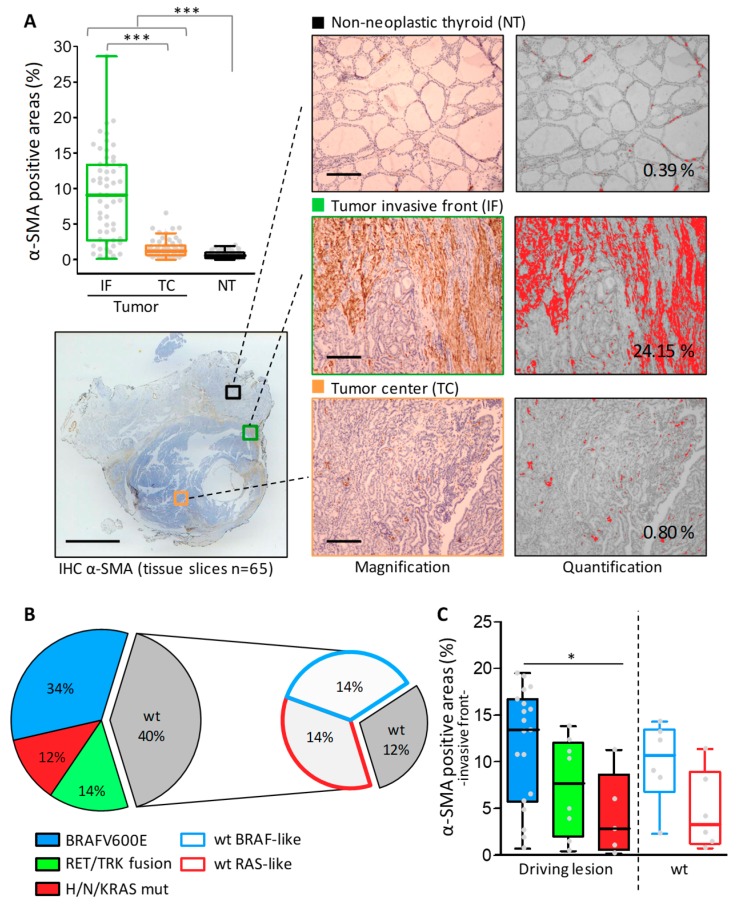
Cancer-associated fibroblasts (CAFs) assessment by α-SMA immunohistochemical staining in human thyroid cancers. (**A**) A boxplot with a scatterplot showing IHC results expressed as α-SMA positive areas calculated by image digital quantification on the stained tissues section from non-neoplastic thyroids (NT) and thyroid tumors (scored separately for invasive front (IF) and tumor center (TC)). Each dot represents the mean of 2–3 fields scored for each sample. Below an example of α-SMA stained tissue section (scale bar: 5 mm) with representative scored regions (high magnification, scale bar: 500 μm) and the corresponding ImageJ threshold mask with the measured positive area (expressed as a percentage). (**B**) Tumor genotyping results. Pie charts represent the distribution of identified driving lesions or BRAF-/RAS-like signaling in tumor tissues; wt: samples with no alterations in the tested lesions. (**C**) Boxplot with scatterplot showing α-SMA IHC results in tumor samples stratified for genetic driving lesion or BRAF-/RAS-like signaling. *** *p*-value < 0.0001 and * *p*-value < 0.05 by Mann-Whitney test.

We found α-SMA positive areas, indicative of an activated fibroblasts presence, in human thyroid tumors (Figure 1A). In particular α-SMA positive areas localized preferentially in the stroma along the tumor invasive front, organized in peripheral structures as fibrotic capsule or septa that were absent in the tumor center and NTs, where α-SMA staining was restricted to blood vessels (Figure 1A, magnification). The extent of stromal α-SMA positive areas, however, was quite heterogeneous ranging from very thick to absent (Figure 1A, α-SMA positive areas in the invasive front ranged from 0.2% to 28%, scored by digital quantification). We therefore wanted to test whether different CAF levels could be associated with specific tumor histotypes (See PTC, PDTC, and ATC in Figure S1). However we did not observe a clear stratification according to this parameter.

Since Jolly et al. [10] reported specific fibroblasts recruitment in murine *BRAFV600E* TCs, we then assessed whether the heterogeneous CAF level could be associated with specific driving lesions, and in particular with *BRAFV600E*, rather than with a tumor histotype.

Some of the tumor tissues reported in this study had been already characterized for driving lesions such as *BRAFV600E* and *H/N/KRAS* mutations, as well as *RET* and *TRK* gene fusions [17]. Tumor genotyping for the same oncogenes was therefore completed for all the samples (Figure 1B and Table S1). Collectively, 42 out of 43 TC patients were screened for genetic alterations. A high fraction (60%) of these had positive results for the tested lesions, with *BRAFV600E* as the most frequently identified alteration. In addition, for a subset of tumors with unidentified lesions (40%, indicated as wild type (wt)), we could exploit gene profiles established on patient matched frozen tissue to investigate their oncogenic signaling. To achieve this aim, we applied the TCGA derived 71-gene signature for BRAF-/RAS-like signaling classification [6] (Figure S2). Accordingly, we confirmed BRAF- and RAS-like signaling in *BRAFV600E* and *RAS* mutated samples, respectively, and obtained signaling information for 70% (12 out of 17) of wt tumors (those with available gene profiles, Table S1), decreasing to 12% of tumors with unidentified lesion or signaling (Figure 1B).

In tumor samples stratified for gene drivers or signaling (Figure 1C), we found not only a higher level of α-SMA staining in *BRAFV600E*- compared with *RAS*-mutated tumors, but also in BRAF-like tumors compared with RAS-like tumors.

Overall, we confirmed that CAFs are enriched at the invasive front of human thyroid cancers and in particular in those harboring *BRAFV600E* mutation or BRAF-like signaling.

### 2.2. CAFs, COL1A1, and LOX Coordinated Expression in Human Thyroid Cancers

We then investigated our tissue series for COL1A and LOX expression, and their possible association with CAFs and BRAF mutation or signaling.

**Figure 2:**
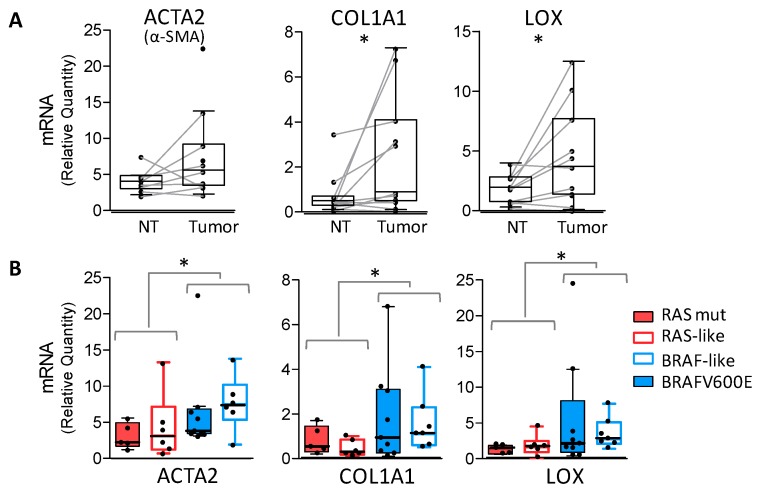
*α-SMA, COL1A1*, and *LOX* gene expression in human thyroid cancers. (**A**) Boxplot with a scatterplot showing mRNA expression of *α-SMA* (*ACTA2* gene), *COL1A1*, and *LOX* genes using quantitative RT-PCR in paired non-neoplastic thyroids (NT) and thyroid tumors. (**B**) The same genes displayed in tumor samples stratified for driving lesion or BRAF-/RAS-like signaling. qRT-PCR data are shown as a relative quantity normalized to the *HPRT* gene used as endogenous control for RNA input normalization. Each dot represents the mean of three technical replicates. * *p*-value < 0.05 using a Wilcoxon. matched test for pairs NT/tumor or the Mann-Whitney test.

We found higher levels of *COL1A1* and *LOX* in tumor tissues compared with NTs (Figure S3A), which resulted in more significant paired tissues (Figure 2A). In the same samples, *α-SMA* gene (*ACTA2*) was also highly expressed in tumors, confirming the presence of IHC staining. Focusing on tumor samples, we did not observe a clear stratification according to the tumor histotype (Figure S3B), even though high *LOX* and *COL1A1* levels were detected in ATCs. In tumors stratified for gene drivers or signaling (Figure 2B), we found significantly higher levels of *ACTA2*, *COL1A1*, and *LOX* genes in *BRAFV600E* or BRAF-like tumors compared with *RAS* mutated or RAS-like tumors.

**Figure 3:**
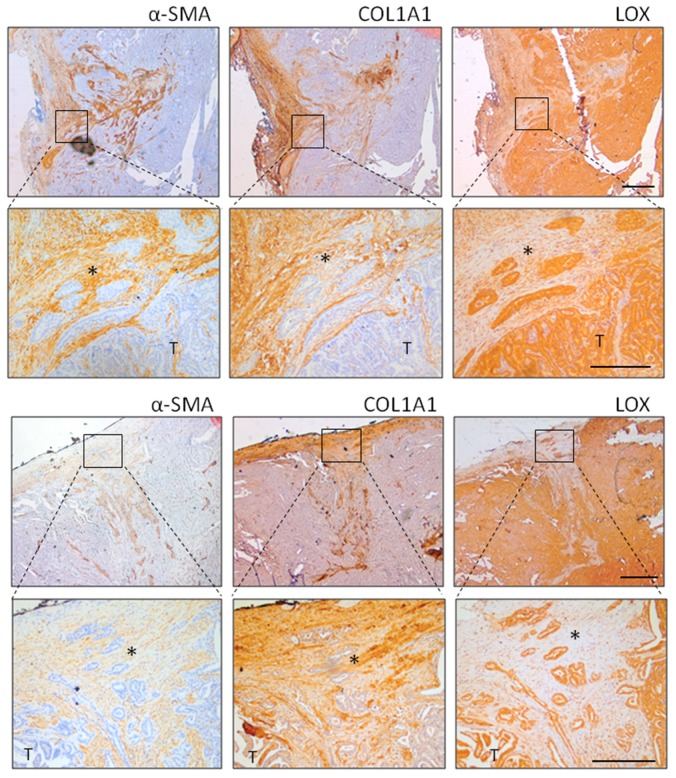
α-SMA, LOX, and COL1A1 immunostaining in human thyroid cancers. Representative thyroid tumors serial sections from two different patients stained by IHC for α-SMA, COL1A1, and LOX protein expression and localization. In the top panel, the tumor edge/invasive front is specifically shown (scale bar 500 µm), while lower panel has higher magnification (scale bar 200 µm). * clusters of tumor invading cells detaching from the principal tumor mass (T).

Immunostaining on tissue serial sections (Figure 3) confirmed the coordinated expression of α-SMA, COL1A1, and LOX. COL1A1 localized in tumor stroma and was closely correlated with α-SMA positive CAFs, whereas LOX was expressed by thyroid tumor cells. In addition, we observed the presence of tumor clusters organized as islands or chords detached from the main tumor mass and infiltrating the connective stroma (Figure 3, higher magnification). This feature of cancer cells of invading as cohesive groups, generally indicated as ‘collective invasion’, was preferentially detected in *BRAFV600E* tumors.

Overall, we showed the concurrent expression of α-SMA, LOX, and COL1A1 genes and proteins in BRAF-driven human thyroid tumors, where we also detected stroma associated local invasion.

### 2.3. Senescent Thyrocytes Are Present at the Invasive Front of Thyroid Cancers and Closely Related to CAFs

As Kim et al. [12] described a similar mechanism of invasion mediated by senescent TC cells in *BRAFV600E* mutated PTCs, we wanted to test whether senescent thyrocytes could be detected at the α-SMA positive and collagen rich stroma interface.

**Figure 4:**
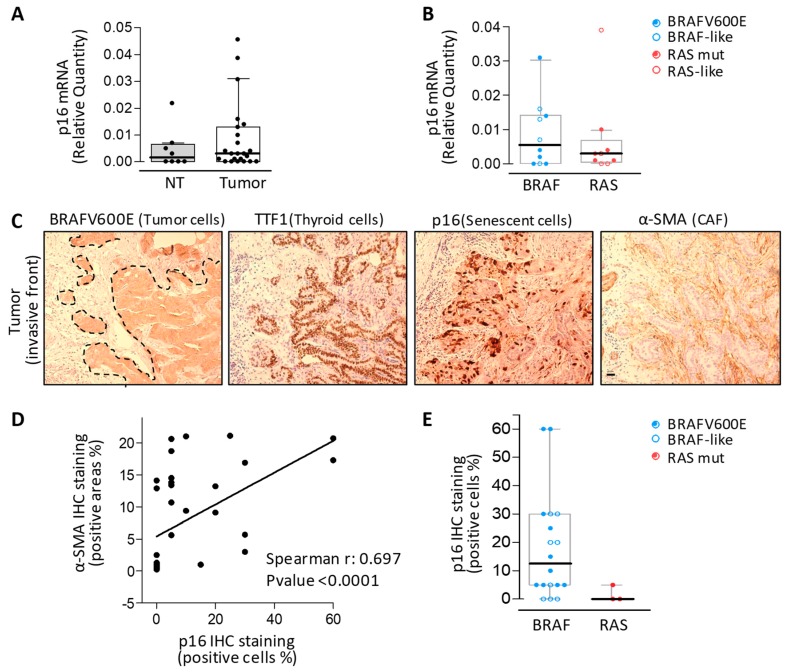
Senescent thyrocytes and CAFs assessment in human thyroid cancers. (**A**) Boxplot with a scatterplot of *p16* mRNA expression by qRT-PCR in non-neoplastic thyroids (NT) and thyroid tumors and (**B**) in tumor samples stratified for driving lesion or BRAF-/RAS-like signaling. qRT-PCR data are shown as a relative quantity normalized to the *HPRT* gene used as an endogenous control. Each dot represents the mean of three technical replicates. (**C**) Representative serial sections of a BRAFV600E mutated thyroid tumor IHC stained with p16 and α-SMA (senescent cells and CAFs markers, respectively). Scale bar: 100 µm. (**D**) Correlation between α-SMA positive areas and p16 positive cells established by IHC. (**E**) Boxplot with a scatterplot of p16 IHC staining results in tumors stratified for driving lesions or signaling.

We examined the expression of p16, a recognized marker for the study of OIS and senescence in vivo. We found higher levels of the *p16* gene in tumor tissues compared to NT, where it was barely detectable (Figure 4A). In tumors stratified for gene drivers or signaling (Figure 4B), we found a trend toward *p16* higher expression in BRAF-driven (*BRAFV600E* or BRAF-like) tumors compared with RAS-driven (*RAS* mutated or RAS-like) tumors, even though this trend was not statistically significant.

We then performed p16 immunostaining on tissue serial sections adjacent to those assessed for α-SMA immunostaining (Figure 4C). We found p16 positive cells in tumor tissues, in agreement with our previous published results [14,15] and localized preferentially at the tumor invasive front (Figure 4C and Figure S4), in agreement with Kim et al. [12]. p16 positive cells were confirmed to be thyroid tumor cells through a morphological assessment and the expression of BRAFV600E (assessed by the BRAFV600E-specific monoclonal antibody) and TTF-1 (the thyroid specific transcription factor-1) (Figure 4C and Figure S4). Cellular senescence was further confirmed by the expression of additional markers, including p21 and Ki67 (cell proliferation marker) (Figure S4). The p21 expression pattern was similar to p16 in localizing at the tumor invasive front, even though less intense staining was observed, and the absence of proliferative activity was further confirmed by the lack of Ki67 expression in p16 and p21 positive cells. Of note, we found that p16 positive cells were closely related (Figure 4C) and significantly correlated with CAFs (α-SMA positive stromal areas, see Figure 4D). Higher levels of p16 positive cells were recorded, in agreement with α-SMA expression (Figure 2B) and IHC staining (Figure 1C), in *BRAFV600E* or BRAF-like tumors compared with in *RAS* mutated tumors (Figure 4E).

Overall, we confirmed the co-occurrence of senescent thyroid cells and CAFs at the invasive front of BRAF-driven human thyroid cancers.

### 2.4. Concurrent Upregulation of LOX, COL1A1, CAFs and Senescent Cells Markers in Thyroid Cancer Gene Profiles

**Figure 5:**
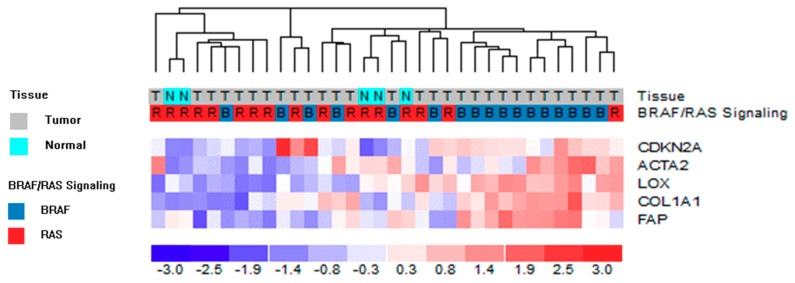
Unsupervised hierarchical clustering analysis of proprietary gene dataset on 5 tumoral and stromal genes. The color scale bar represents the relative gene expression levels normalized by the standard deviation. Color legend for tissue and BRAF-like/RAS-like signaling is reported. *α-SMA* and *p16* are indicated by the corresponding gene symbol, *ACTA2* and *CDKN2A*, respectively.

Solid tumors consist of both tumor cells and TME, including stromal cells. As a consequence, gene profiles obtained from such complex tissues are a mixture of RNA transcripts derived from all of these components. We wanted to assess the concurrent expression of genes detected in both tumoral (LOX and p16) and stromal compartments (α-SMA and COL1A1). We exploited the transcriptomic profiles available for a subset of tissues of our series (Table S1) and investigated the expression of *α-SMA*, *COL1A1*, *LOX*, and *p16* genes. We included the *FAP* gene (fibroblast activation protein, a well known activator of CAFs specifically expressed by stroma [18]) as an additional marker for CAFs (Figure 5). In line with the so far described results, we confirmed the concurrent high expression of both tumoral and stromal derived genes in tumor tissues and in particular in those characterized by BRAF-like signaling (11 out of 17 BRAF-like tumors clustered in the 5-genes overexpressing group; *p*-value 0.0032).

To further confirm this co-occurrence, we interrogated additional thyroid tissue gene datasets that are publicly available on the GEO repository.

**Figure 6:**
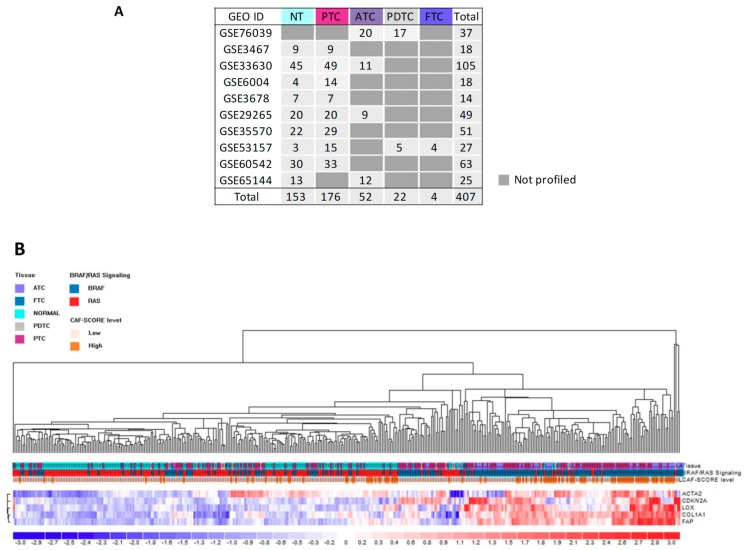
Assessment of tumoral and stromal derived genes in human thyroid cancers gene sets derived from Gene Expression Omnibus (GEO). (**A**) Gene sets description. (**B**) Unsupervised hierarchical clustering analysis of 407 samples derived from GEO datasets on five tumoral and stromal genes. The color scale bar represents the relative gene expression level normalized by the standard deviation. Color legend for the tissue, BRAF-like/RAS-like signaling, and CAF score is reported. α-SMA and p16 are indicated by the corresponding gene symbol, ACTA2 and CDKN2A, respectively.

Ten gene sets were selected (details in Figure 6A). Taking advantage of the fact that all sets were profiled by the same microarray platform, we could analyze the samples as a unique series of 407 thyroid tissues, including 254 TCs of different histotypes, 146 paired NTs, and seven unpaired NT controls. Since detailed information about driving lesion were available only for a fraction of tumors (113 out of 254 TCs, 44%; Table S2), we exploited the TCGA-derived 71 gene signature [6] to infer their BRAF- or RAS-like signaling (Figure S5). By using this approach, we not only confirmed BRAF-/RAS-like signaling in samples with identified drivers, but also obtained signaling information in tumors with unknown or not available lesions (69/254 (27%) and 72/254 (29%), respectively; see Table S2). Of note, in agreement with a previous report [7], we found in the GEO derived sample series (Figure S5), as well as in our proprietary series (Figure S2), that non-neoplastic thyroids display RAS-like signaling, suggesting how this feature could be not exclusively associated to oncogenic RAS but rather to MAPK pathway reduced activation that is also observed in normal thyroid follicular cells. Unsupervised clustering confirmed the target genes (*LOX*, *p16*, *α-SMA*, *COL1A1*, and *FAP*) upregulation in thyroid cancers and in particular in those with BRAF-like signaling (115 out of 206 BRAF-like tumors clustered in the 5-genes overexpressing group; *p*-value < 0.0001) (Figure 6B).

In addition, recently Isella et al. [19] have described gene signatures that are useful for assessing the contribution of stromal components, including CAFs, in tissue transcriptional profiles. In particular they reported a 131 genes signature, specifically expressed by CAFs, that can be exploited to calculate a CAF score indicative of the presence of stromal CAF. Based on this gene signature, we calculated the CAF score in the same GEO derived series and verified that the fraction of BRAF-like tumors displaying upregulation of *LOX*, *COL1A*, CAFs, and senescent cells markers is concurrently enriched for a high CAF score (Figure 6B).

Overall, these results confirm the concurrent gene upregulation of *LOX*, *COL1A1*, senescent cells, and CAFs markers in tumor tissues and in particular in those with BRAF-like signaling.

## 3. Discussion

Previous reports have separately investigated the functional role of cancer-associated fibroblasts [10] and senescent TC cells [12] in the progression of *BRAFV600E* driven PTC. In this study, we exploited a combined approach of immunohistochemistry and gene expression analyses, coupled with tumor genotyping, to simultaneously investigate the occurrence of these two cell types in a proprietary series of human thyroid cancers, as well as in multiple public gene expression datasets.

We found stromal CAF recruitment at the tumor invasive front associated with collagen deposition and LOX expression by the adjacent thyroid tumor cells. In addition, we detected clusters of invading tumor cells at the CAFs and collagen rich tumor-stroma interface where senescent TC cells were also identified, displaying significant co-occurrence with CAFs.

While the cross-talk of CAFs-TC cells has been already confirmed in functional analyses [10] and here in human tissues, how CAFs are recruited and/or activated in the tumor stroma remains to be established. In this scenario, senescent cells represent an attractive candidate for their ability of affecting surrounding cells and TME by the secretion of multiple factors (SASP). Our results, describing the co-occurrence of CAFs and senescent TC cells at the tumor-stroma interface, are in line with this hypothesis and suggest senescent TC cells as a possible source of secreted factors involved in the recruitment and/or activation of stromal fibroblasts. In turn CAFs, through a concerted action with thyroid tumor cells, lead to extracellular matrix stiffness through the deposition and LOX mediated cross-linking of collagen fibers, as shown by Jolly et al. and here confirmed in human tissues. In addition, our results show how the collagen-rich stroma appears to draw a privileged route by which clusters of tumor cells could migrate detaching from the main tumor mass as a first step toward local invasion.

While Kim et al. demonstrated the role of senescent TC cells in the invasive phase, where they lead TC cells migration [12], their possible early role on CAF recruitment/activation, as well as the secreted mediators involved, remain to be verified and future functional studies will be conducted to investigate this issue. To the best of our knowledge, no studies about senescent TC cells and CAFs are currently available. Some studies have assessed the interactions between senescent cells and other cell types as platelets [20] and immune cells [21]. In addition, our laboratory has recently described an interplay between senescent thyrocytes and macrophages [22]. Several works (reviewed in reference [16]) describe the presence of senescent fibroblasts along with CAFs in tumor stroma and how both can promote tumor aggressive features. In our IHC analyses, we only identified few patients harboring senescent fibroblasts, while senescence was detected in tumor cells in the vast majority of patients, suggesting that the cellular type undergoing to cellular senescence could vary based on the specific tumor context.

However, some evidence suggests that there is a possible cross-talk between senescent cells and CAFs. It is known that tumor cells release paracrine factors able to attract and/or activate CAFs [16]; for instance, transforming growth factor (TGF)-β1 [23], interleukin (IL)-1, and IL-6 [24] have been reported in the activation of fibroblasts. Accordingly, tumor cells expressing TGF-β1 have been associated with α-SMA fibroblasts in PTC [25]. Interesting, these factors were also identified in senescent thyroid cells. TGF-β1 [22], IL-1, and IL-6 [12,15,22] are expressed by two thyroid models of oncogene-induced senescence, as well as by senescent TC cells at the invasive border of human PTCs [12]. It is noteworthy that we found that the same mediators (*TGF-β1*, *IL-1* and *IL-6*) are expressed by tumors with high *LOX*, collagen, CAFs, and senescent cells markers (Figure S6), supporting the hypothesis of a possible cross-talk between CAFs and senescent TC cells. In line with CAF activation mediated by thyroid tumor cells, Jolly et al. [10] reported that *BRAFV600E* TC cells, derived from their mouse model, secrete factors that are able to promote the proliferation and migration of two CAF cell lines. Jolly et al. did not assess whether part of these tumor cells expressed senescence markers, but it is interesting that the secretome of *HRAS*-mutated thyroid tumors cells did not cause these stimulatory effects, consistently with our findings that CAFs and senescence markers do not often co-occur in RAS-driven human thyroid tumors.

In addition to PTC, we investigated CAFs in aggressive forms as PDTC and ATC. However, we did not see a clear correlation with specific histotypes (Figure S1), but instead we found an association with tumor genotype showing higher levels of CAFs in BRAF-driven tumors (Figure S7A). This finding could be interpreted in light of the notion that some genetic alterations (such as *BRAFV600E* and *RAS* mutations) even though specific, or at least more frequent, in certain TC histotypes (papillary versus follicular) are commonly shared among well differentiated and poorly or undifferentiated forms. As PDTC and ATC may arise from both PTC and FTC, they also can be driven, as their corresponding WDTC of origin, by BRAF/MAPK or RAS/PI3K-AKT pathway alterations, thus displaying genotypic and phenotypic differences. This was also confirmed in public GEO datasets (Figure 6), where tumors displaying high target genes levels are BRAF-driven PTCs and ATCs, while most of the PDTCs of the series, characterized by RAS-like signaling, downregulate the same genes. The proposed model in which CAFs-TC cells cross-talk promotes the progression from PTC to aggressive PDTC [10] thus requires a specification: this interaction is accordingly observed in human thyroid tumors, both in PTC and aggressive PDTC or ATC, but it appears to be distinctive for BRAF-driven tumors. In line with the idea of a linear progression from less to more advanced forms, we found increased expression of *α-SMA*, *COL1A1*, and *LOX* in the more aggressive tumor component (ATC versus PTC or PTC tall cell versus a classical variant) in two TC patients of our series for whom a tumor with a mixed component or multiple tumor specimens were available (Figure S7B). This finding, however, needs to be verified in additional and large case lists possibly comprising patient matched tumor tissues with various degrees of aggressiveness.

Our results suggest the possibility that CAFs and TC cells may interact to promote tumor local invasion. Only a few previous studies have investigated CAFs in human thyroid tumors and were mainly focused on PTC, in which the occurrence of CAFs has been accordingly reported in tumor stroma [25,26,27,28,29] and associated with aggressive features as advanced stage [25] and lymph node metastases (LNMs) [26,28]. The presence of fibrotic collagenous stroma has been significantly associated with LNMs and peritumoral invasion in papillary thyroid microcarcinomas [30]. In addition, increased expression of CAF-related proteins was significantly found in stromal cells of classical compared to the follicular variant of PTC and in *BRAFV600E* mutated PTCs, where a tumor infiltrative margin was also detected [27].

Interestingly, Kim et al. [12] reported that senescent TC cells can promote not only local invasion, but also LNMs, identified in more than half (61%) of the investigated *BRAFV600E* PTC patients. The correlation between *BRAF* mutation and aggressive features, including LNMs, has been already described not only in PTC, but also in aggressive forms. Landa et al. [5] reported that *BRAF*-mutant PDTCs had a higher frequency of nodal metastases than *RAS*-mutant PDTCs that, instead, displayed a higher rate of distant metastases. 

This appears to be in line with an idea of genotype-phenotype correlation: alterations involving *BRAF* or other genes that activate the MAPK pathway (for instance, several *RET* gene fusions reported in reference [6] and here confirmed displaying BRAF-like signaling see Tables S1 and S2) are more frequently detected in TCs of papillary origin (PTC and PDTC/ATC that derive from PTC) that display more frequent metastatic spreading through lymphatic vessels and LNMs. Meanwhile, alterations involving *RAS* or other genes (*PAX8/PPARG*, specific gene fusions and PI3K pathway effectors) that display RAS-like signaling are more frequently detected in TCs of follicular origin (FTC and PDTC/ATC that derive from FTC) that display more frequent spreading via blood vessels and distance metastases.

It is conceivable, therefore, to speculate that the TC invasiveness, observed here in the presence of both CAFs and senescent cells, could be not limited to local invasion but possibly could also expand to LN dissemination, in line with Kim et al.’s model [12]. To test this hypothesis, we assessed whether tumors with high *LOX*, collagen, CAFs, and senescent cells markers could also be associated with lymph node metastases. In our series, we consistently found that nine out of 12 tumors with high expression of the target genes (Figure 5) had LNMs. For the remaining three tumors, an LN assessment and status was not available (Table S1, NX). In the series derived from GEO, this analysis was not applicable as records about LNs were limited. We, therefore, assessed the series of PTCs derived from TCGA [6] (available on www.cbioportal.org with complete clinicopathological records) and confirmed that PTCs with coordinated high expression of the tested genes (*LOX*, *COL1A1*, *p16*, *ACTA2* and *FAP*) were also significantly associated with LNMs (Figure S8), in agreement with our hypothesis.

Even though additional functional studies are required to address some open questions, we believe that the present study, showing how CAFs and senescent TC cells are both present at the tumor invasive front of BRAF-driven TCs, highlights that they represent additional players worthy of being studied for a better understanding of the metastatic process in TC.

## 4. Materials and Methods

### 4.1. Tissue Samples Collection and Nucleic Acid Extraction

A retrospective series of non-consecutive human thyroid tumors including PTCs, PDTCs, and ATCs was investigated in the present study, and clinicopathological features are summarized in Table S1. Tumor tissues were collected from patients diagnosed with thyroid carcinoma and undergoing surgical resection. Whether paired non-neoplastic thyroid tissue was available from the same patient, it was also investigated. Additional normal thyroid controls from patients with pathologies other than thyroid cancer were included. Tissue samples were obtained from the Department of Pathology at Fondazione IRCCS Istituto Nazionale dei Tumori, Milan (INT). The study was approved by the Independent Ethics Committee of INT (code number INT 55/14). Formalin-fixed paraffin-embedded (FFPE) tissue blocks were cut in serial sections and processed for histological review, DNA and RNA extraction, and immunohistochemistry (IHC). Histological review was performed by expert pathologists on hematoxylin and eosin (H&E) stained sections and tumor tissues were classified according to WHO Classifications [31] and using the pathological tumor-node-metastasis (pTNM) staging system [32].

DNA was extracted from FFPE tumor tissues. Methylene blue-stained sections (7 µm) were microdissected to select tumoral and non tumoral tissues, while all tumoral sections contained at least 80% of a neoplastic component. Tumor genomic DNA was extracted using the Qiamp FFPE DNA kit (Qiagen, Hilden, Germany) and was used for tumor genotyping.

RNA was extracted from manually macrodissected unstained sections (5 µm) using adjacent H&E slides as a guide for whether matched NT tissue was available from the same section and could be extracted as a separate sample. RNA was extracted by miRNeasy FFPE kit (Qiagen) and used for gene fusion detection in tumor genotyping and for qRT-PCR analyses.

A subset of patients, based on frozen tissue availability, were further investigated by gene expression profiling. H&E stained sections from snap-frozen tumoral samples were reviewed by an expert pathologist for tissue representativeness control and were subjected to RNA extraction (see Section 4.5).

### 4.2. Tumor Tissue Genotyping

Tumor samples were screened for some of the most common thyroid oncogenes, including BRAF and N/H/KRAS mutations as well as RET and TRK gene fusions. *BRAF* (exon 15), *N/HRAS* (exon 2), and *KRAS* (exon 2 and 3) mutational analysis was performed on tumor genomic DNA by using PCR specific primers that were previously described [33,34]. PCR products were then subjected to direct sequencing using 3500 DX Genetic Analyzer (Applied Biosystems, Thermo Fisher Scientific, Waltham, MA, USA) and evaluated by using ChromasPro software. Gene fusions analysis was performed on tumor RNA reverse transcribed in cDNA and assessed by RET and TRK specific primers as previously described [17]. Samples that were not mutated or rearranged for the tested genes were indicated as being of the wild type (wt).

### 4.3. Immunohistochemistry

IHC analyses were performed on FFPE tumor sections (2 µm) by means of an automated immunostaining device (Autostainer link48 Dako, Agilent, Santa Clara, CA, USA) using anti-human α-SMA (actin smooth muscle, clone 1A4, M085101-2, 1:800, Dako) and anti-TTF1 (#86763, 1:2000, Dako). Immunostaining of COL1A1 (NB600-408, 1:400, Novus Biologicals, Centennial, CO, USA), p16 (p16^INK4a^, prediluted, CINtec^®^, Roche, Basel, Switzerland), and BRAFV600E (06918727001, prediluted, Roche) was performed on Benchmark ULTRA IHC instrument (Ventana, Roche). LOX expression was assessed by using heat-induced epitope retrieval (pressure cooker) and immunostaining with anti-LOX (NB100-2530 1:400, Novus Biologicals).

The assessment of α-SMA immunostaining was performed by digital quantification using ImageJ software. For each tissue section representative images of different regions (tumor center, tumor invasive front and, whether available, adjacent non-neoplastic thyroid) were captured (Nikon ECLIPSE E600, Nikon Corporation, Tokyo, Japan), with two or three fields acquired per region. As α-SMA immunostains also contained blood vessels pericytes, to avoid quantification artifacts, fields were selected in areas with negative vascular structures or harboring, at least, very low vascularity (less than 3% of the scored field). Images were thresholded and processed by using the analyze-measure tool in ImageJ setting as the output for the area fraction measurement (indicative of the stained areas percentage relative to the total field).

The assessment of p16 immunostaining was determined as number of p16 positive tumor cells relative to the total tumor cells in the tissue slice expressed as a percentage.

### 4.4. Quantitative Real-Time Reverse Transcription PCR (qRT-PCR)

The expression of *LOX*, *COL1A1*, *αSMA* (*ACTA2* gene), and *p16* (*CDKN2A* gene) was determined by a two-step quantitative real-time PCR on RNA extracted from FFPE tissue slices. RNA was reverse transcribed by SuperScript III First-Strand Synthesis System for RT-PCR (Invitrogen, Thermo Fisher Scientific) and quantified by TaqMan Universal PCR Master Mix and Gene Expression Assays (LOX hs00942483_m1; COL1A1 hs01076773_g1; ACTA2 hs00909449_m1; CDKN2A hs99999189_m1) (Applied Biosystems, Thermo Fisher Scientific). *HPRT* gene (assay hs02800695_m1) was used as an endogenous control for data normalization. All qRT-PCRs were performed in triplicate on the ABI PRISM 7900HT Real-Time PCR System. Data were analyzed with SDS 2.4 and RQ Manager 1.2.1 software (Applied Biosystems) using the 2^−ΔΔCt^ method.

### 4.5. Gene Expression Profiles-Proprietary Series

RNA was extracted from snap-frozen tissues and quantified by NanoDrop ND-10 Spectrophotometer (NanoDrop Technologies, Wilmington, DE, USA) as previously described [17]. RNA quality was evaluated by the RNA Integrity Number (RIN) assessed using Agilent 2100 Bioanalyzer (Agilent Technologies, Palo Alto, CA, USA), since the obtained RNA was of a variable quality (mean RIN 4.9, range 1.8–7.8) gene profiles were established by HumanHT-12 WG-DASL V4.0 R2 expression beadchip (Illumina, San Diego, CA, USA), and was optimized to ensure high performance from difficult or partially degraded RNA samples. RNA labeling, processing, and hybridization were performed according to Illumina standard protocols, and microarrays were scanned with a Illumina BeadArray Reader. Array data were obtained using Illumina BeadStudio v3.3.8 and processed using the lumi package [35]. Raw data were log2-transformed, normalized using robust spline normalization, and filtered keeping probes with a detection *p*-value < 0.01 in at least one sample. Probes were annotated to HUGO gene symbols using the illuminaHumanWGDASLv4.db package (Illumina). Multiple probes representing the same gene were collapsed using the collapseRows function of the WGCNA package [36] with the “maxRowVariance” method. All analyses were performed using R version 3.3.0 and Bioconductor [37] version 3.4. Microarray data were deposited and are available on NCBI Gene Expression Omnibus (GEO) database (www.ncbi.nlm.nih.gov/geo/) with the accession number GSE104005. Part of the expression profiles of this series has been already published [17].

The BRAF-/RAS-like signaling and *LOX*, *COL1A1*, *αSMA* (*ACTA2* gene), *p16* (*CDKN2A* gene), and *FAP* genes expression were investigated in this series as described below.

### 4.6. Gene Expression Profiles-Public Datasets

Ten human thyroid cancer gene sets publicly available in the GEO repository (GSE3467 [38], GSE6004 [39], GSE33630 [40,41], GSE76039 [5] GSE35570 [42], GSE65144 [43], GSE53157 [44], GSE29265, GSE3678, and GSE60542 [45]) and profiled on the same microarray platform (Human Genome U133 Plus 2.0 Array, Affymetrix, Santa Clara, CA, USA) were used in the present study. For the GSE35570, series we excluded 65 samples as duplicated in GSE33630 from the same laboratory. For the GSE60542 series, we focused on primary PTCs and paired NTs. A single series of 407 unique cases was obtained, thus comprising 176 PTC, 52 ATC, 22 PDTC, and 4 FTC, together with 153 normal thyroid samples. Robust Multi-array Average (RMA) normalization [46], BrainArray annotation (http://brainarray.mbni.med.umich.edu/, version 20.0.0) [47] and batch correction by sva R/Bioconductor package [48] were applied to raw intensity values, as previously described [49].

In order to investigate the expression of *LOX*, *COL1A1*, *αSMA* (*ACTA2* gene), *p16* (*CDKN2A* gene), and *FAP* genes across the entire dataset, a hierarchical clustering approach was performed using the euclidean distance metric and centroid linkage method, by means of the DNAChip Analyzer [50].

### 4.7. BRAF-Like and RAS-Like Signaling

The BRAF-/RAS-like signaling was investigated in 407 samples dataset by exploiting the TCGA-derived 71 genes signature [6]. The corresponding BRAF- or RAS-like molecular group was attributed to each sample by using hierarchical clustering analysis (Pearson’s correlation metric and average linkage method) on the global 71-gene list expression pattern across the entire series (Figure S5). The same kind of analysis was performed on the proprietary dataset using 68 genes out of the 71-gene signatures available on Illumina array (Figure S2).

### 4.8. CAF Score

The CAF score was attributed to all 407 samples by using the 131 genes signature previously reported by Isella et al. [19]; of the 131 genes, 125 were available on HG-U133 Plus 2.0 array and were used to compute the CAF score. The 125 genes average expression value was calculated for each sample and the entire dataset was classified in CAF score quartiles and further in low (first to third quartile) or high (top quartile) CAF score level.

### 4.9. Statistical Analysis

Statistical analyses were performed using GraphPad Prism software (version 5.02, GraphPad Software, San Diego, CA, USA). Groups were compared using the non parametric Mann-Whitney test or Wilcoxon matched pairs test for tumor/non-neoplastic thyroid matched samples; *p*-values < 0.05 were considered to be significant. Correlation was tested by the non parametric Spearman correlation coefficient (r).

## 5. Conclusions

The analyses on human thyroid cancer tissues presented in this work provide lines of evidence that not only confirm previous reports about the involvement of cancer-associated fibroblasts and senescent TC cells in BRAFV600E-expressing PTC, but also provide a revised and improved model of thyroid cancer invasion in which these two cell types may possibly cooperate to promote the invasiveness of other thyroid cancers driven by BRAF-associated oncogenic events.

## Supplementary Materials

The following are available online at https://www.mdpi.com/2072-6694/12/1/112/s1, Figure S1: CAFs assessment by α-SMA IHC staining in human thyroid cancers stratified for tumor histotype, Figure S2: Unsupervised hierarchical clustering of proprietary gene dataset on the TCGA derived BRAF-/RAS-like signature, Figure S3: α-SMA, LOX and COL1A1 gene expression in human thyroid cancers by quantitative RT-PCR, Figure S4: Assessment of senescence markers by IHC staining in human thyroid cancers, Figure S5: Unsupervised hierarchical clustering of 407 samples derived from GEO datasets on the TCGA derived BRAF-/RAS-like signature, Figure S6: Unsupervised hierarchical clustering analysis of GEO derived and proprietary gene datasets on CAF activating factors, Figure S7: α-SMA, LOX and COL1A1 gene expression by quantitative RT-PCR in matched tumor tissues, Figure S8: Unsupervised hierarchical clustering of 486 PTCs from TGCA dataset and lymph node metastases correlation, Table S1: Human thyroid tumors clinicopathological features, Table S2: Driving lesion and BRAF-/RAS-signaling in the 254 tumor samples from GEO datasets.
