# Supplementary material for: Cancer Associated Fibroblasts and Senescent Thyroid Cells in the Invasive Front of Thyroid Carcinoma
Source: Cancers (Basel). 2020 Jan 1;12(1):112. doi: 10.3390/cancers12010112 (PMC7016563; doi:10.3390/cancers12010112)
Supplement: Supplementary file 1 [file cancers-12-00112-s001.pdf]

Supplementary Materials: Cancer Associated Fibroblasts and Senescent Thyroid Cells in the Invasive Front of Thyroid Carcinoma

Emanuela Minna, Silvia Brich, Katia Todoerti, Silvana Pilotti, Paola Collini, Elisa Bonaldi, Paola Romeo, Loris De Cecco, Matteo Dugo, Federica Perrone, Adele Busico, Andrea Vingiani, Ilaria Bersani, Andrea Anichini, Roberta Mortarini, Antonino Neri, Giancarlo Pruneri, Angela Greco and Maria Grazia Borrello

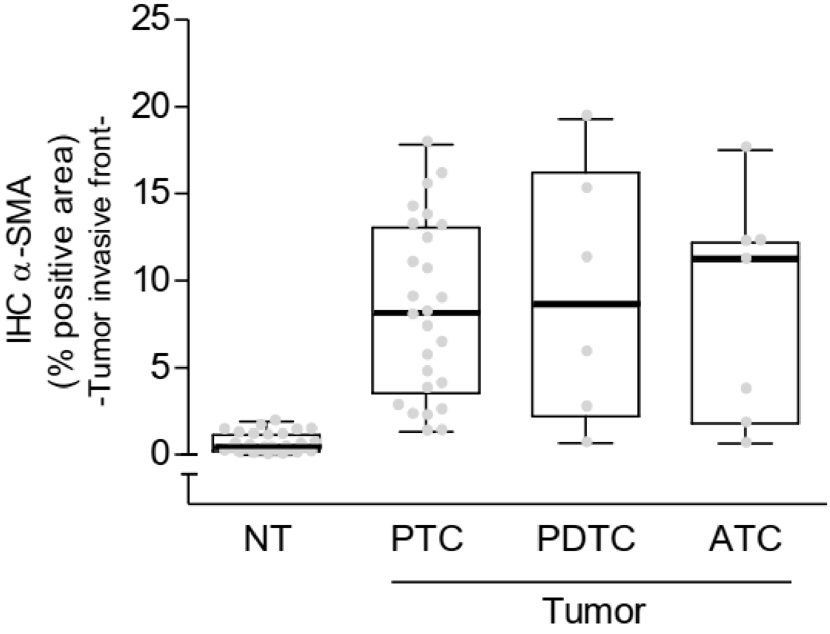

Figure S1. CAFs assessment by α-SMA IHC staining in human thyroid cancers stratified for tumor histotype. Boxplot with scatterplot showing IHC results expressed as α-SMA positive areas calculated by image digital quantification. Each dot represents the mean of 2-3 fields scored for samples; for tumor samples the results relative to invasive front are specifically showed. NT, non-neoplastic thyroid; PTC, papillary thyroid carcinoma; PDTC, poorly differentiated thyroid carcinoma; ATC, anaplastic thyroid carcinoma.

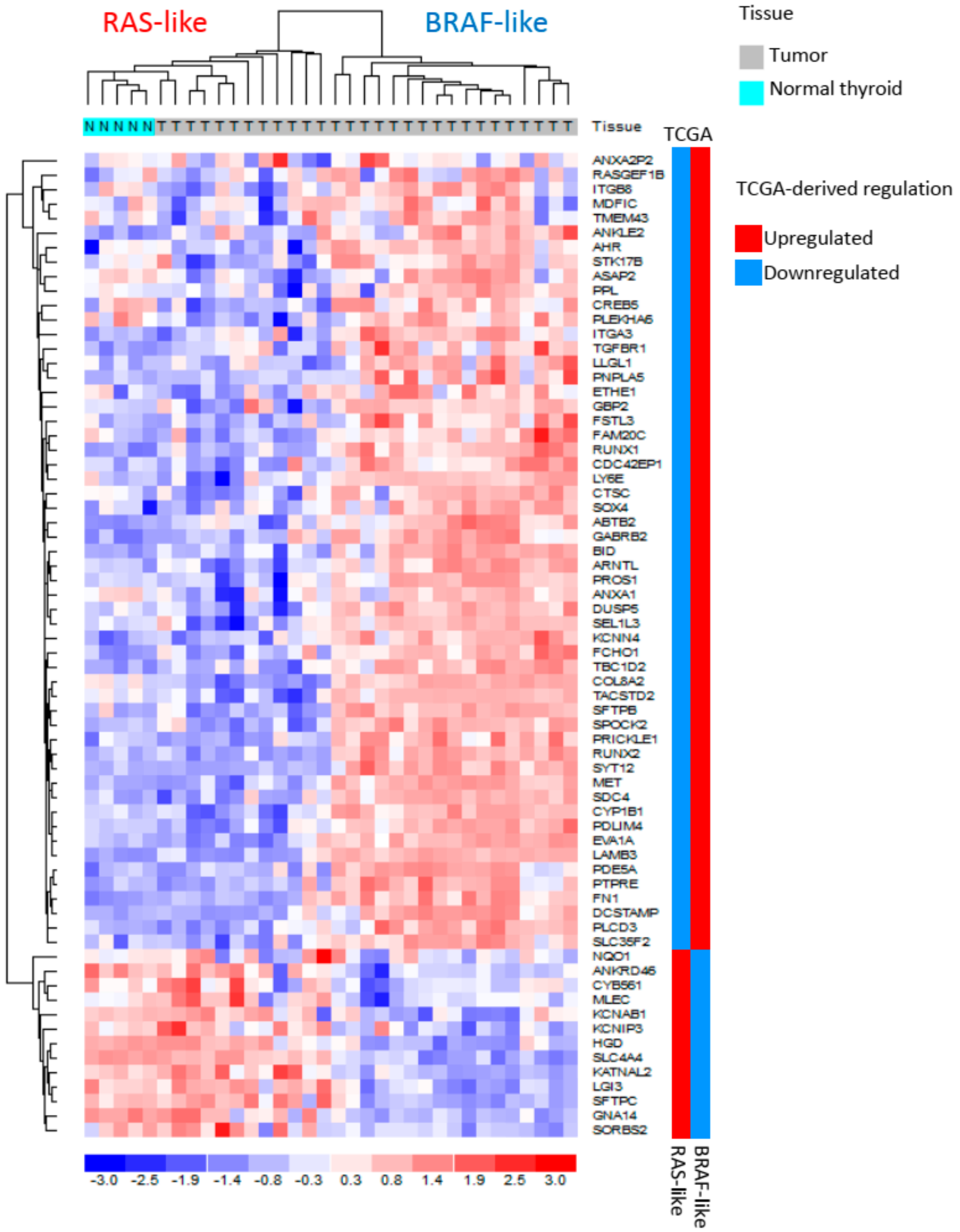

Figure S2. Unsupervised hierarchical clustering analysis of proprietary gene dataset on the TCGA derived BRAF-/RAS-like signature, using pearson's correlation metric and average linkage method. The color scale bar represents the relative gene expression level normalized by the standard deviation. Color legend for tissue type is reported. The vertical bars on the right show the direction of regulation reported by TCGA: red lines show upregulated genes and blue lines show downregulated genes. According to TCGA classification: BRAF-like tissues display signature upregulation, indicative of MAPK pathway transcriptional activation, with only 13 out of the 71 genes

downregulated; RAS-like tissues display signature downregulation, with only the same 13 genes upregulated. Two main clusters are identified with expression pattern consistent with BRAF- or RAS-type. The BRAF-/RAS-like signaling class was attributed to our tissues based on BRAF-/RAS- clustering classification.

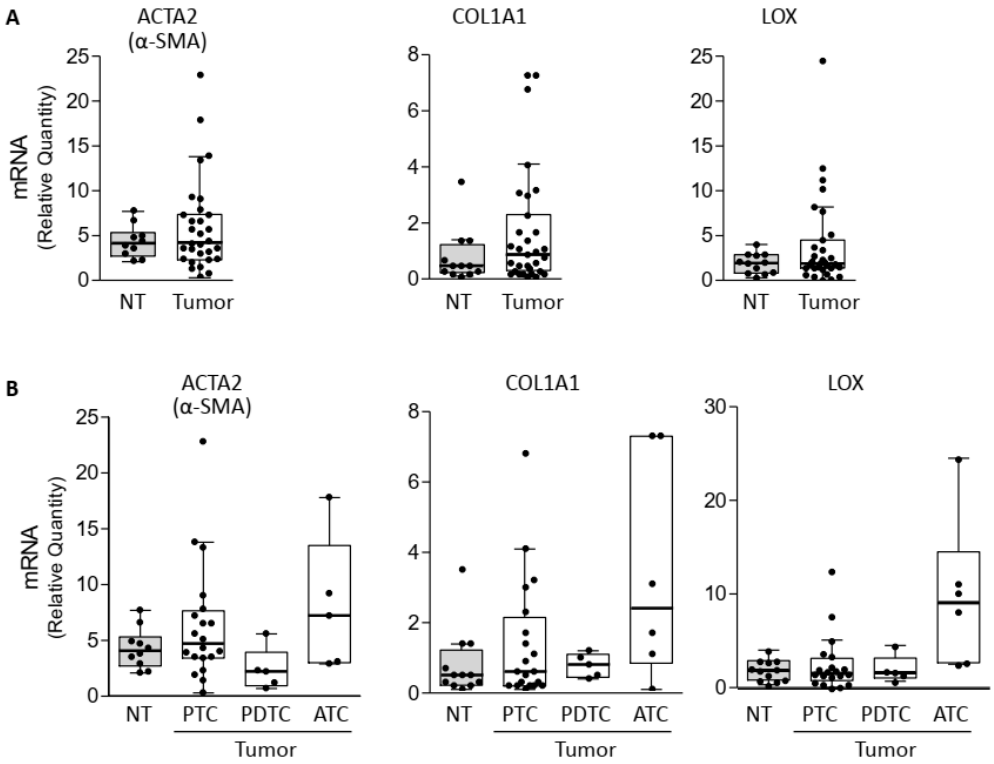

Figure S3. *α-SMA*, *COL1A1* and *LOX* gene expression in human thyroid cancers by quantitative RT-PCR. Boxplot with scatterplot showing mRNA expression of *α*-SMA (*ACTA2* gene), *COL1A1* and *LOX* genes in (**A**) non- neoplastic thyroids (NT) and thyroid tumors and in (**B**) tumors stratified for histotype. qRT-PCR data are shown as relative quantity normalized to HPRT gene used as endogenous control for RNA input normalization. Each dot represents the mean of 3 technical replicates.

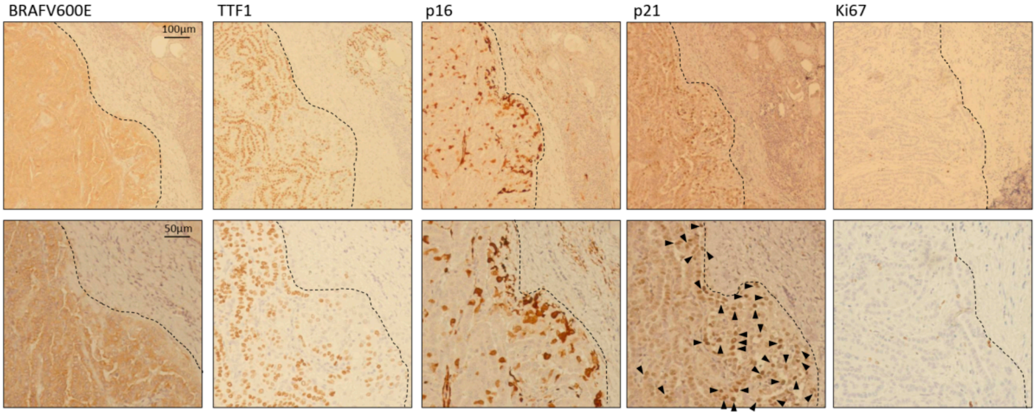

Figure S4. Assessment of senescence markers by IHC staining in human thyroid cancers. Representative images of a tumor serial sections immunostained for the expression of BRAFV600E (marker for BRAFV600E mutated thyroid tumor cells), TTF1 (marker for thyroid cells), p16(alias INK4a) and p21(alias CIP1) (cell cycle inhibitors; markers for cellular senescence) and Ki67 (cell proliferation marker). The area of the invasive front is highlighted by dashed lines and is specifically shown. Two different magnification are shown. Black triangles indicate representative p21 positive nuclei. Immunostaining was performed as described in Materials and Methods; anti-p21 (C-19: sc-397, Santa Cruz Biotechnology) and anti-Ki67 (MIB1 clone, Dako) were used for the additional markers.

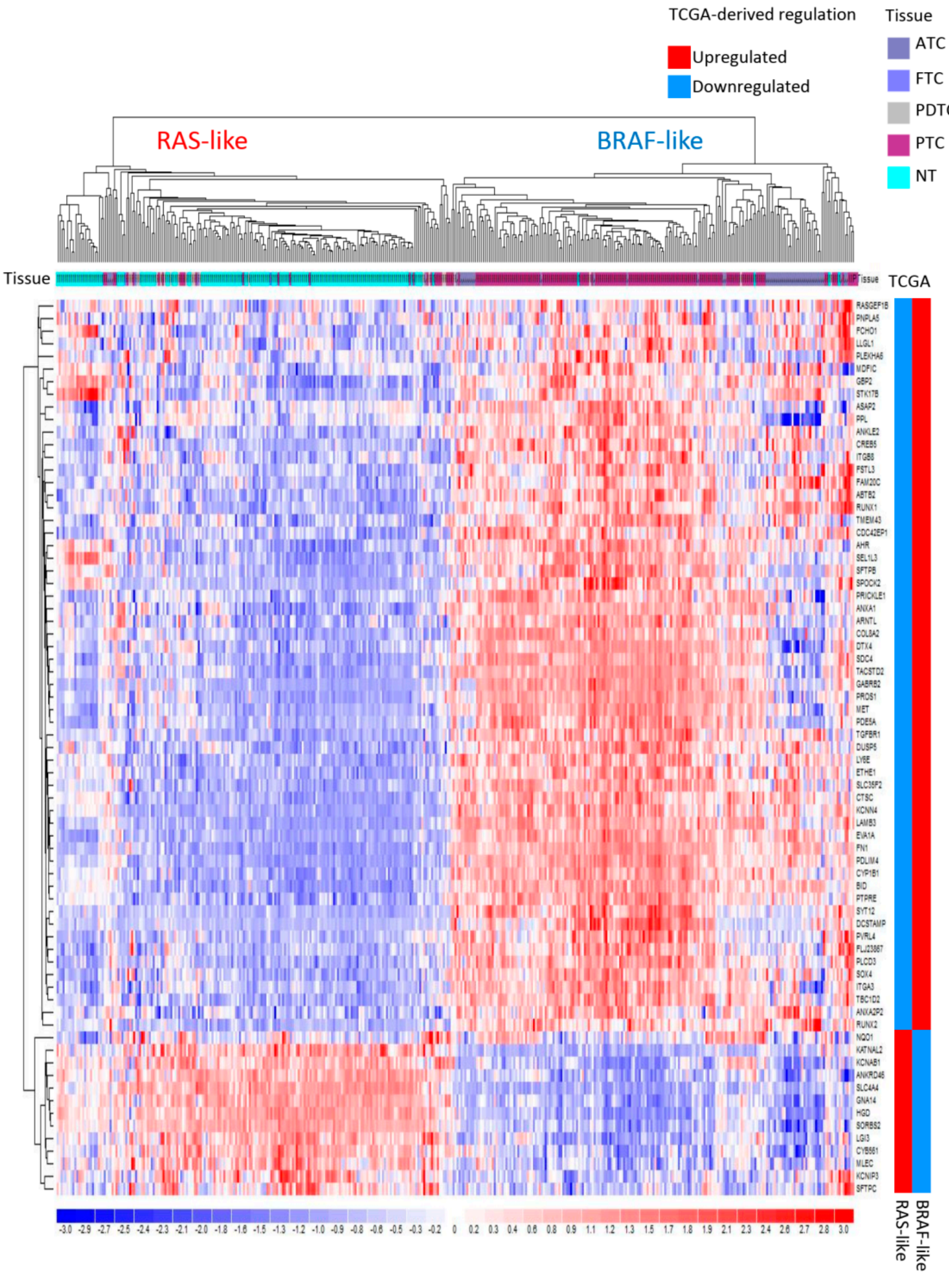

Figure S5. Unsupervised hierarchical clustering analysis of 407 samples derived from GEO datasets on the TCGA derived BRAF-/RAS-like signature, using pearson's correlation metric and average linkage method. The color scale bar represents the relative gene expression level normalized by the standard deviation. Color legend for tissue type is reported. The vertical bars on the right show the direction of regulation reported by TCGA: red lines show upregulated genes and blue lines show downregulated genes. The BRAF-/RAS-like signaling class was attributed based on BRAF-/RAS- clustering classification.

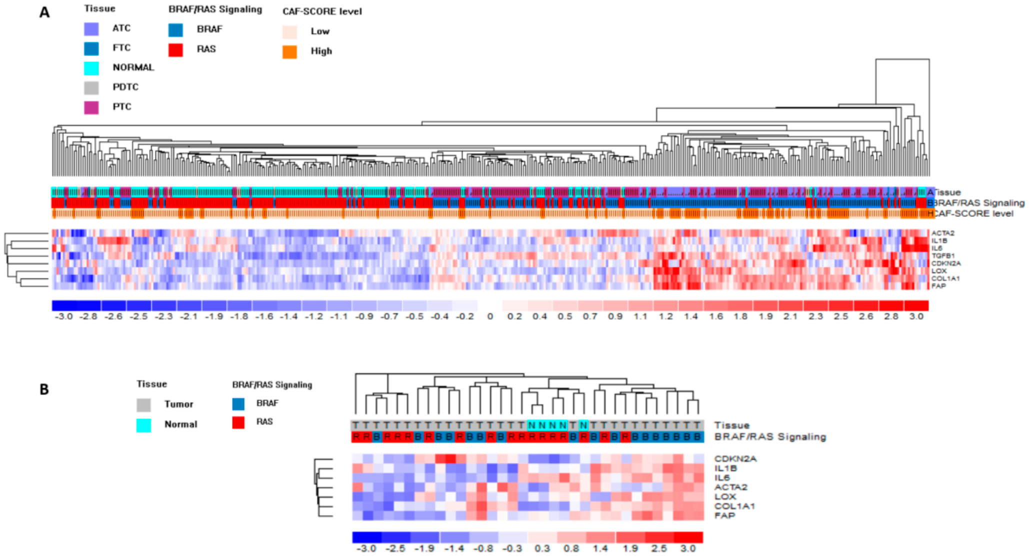

Figure S6. Unsupervised hierarchical clustering analysis of GEO derived and proprietary gene datasets on CAF activating factors. The expression of three factors (*TGF-β1*, *IL-1* and *IL-6*) reported in the activation of CAFs is showed concurrently with *LOX*, *COL1A1*, CAF (*FAP* and *α-SMA* –alias *ACTA2*-) and senescent cells (*p16* –alias *CDKN2A*-) markers in the 407 samples derived from GEO datasets (**A**) and in the proprietary gene dataset (**B**); in this latter only *IL-1* and *IL-6* expression data were available. The color scale bar represents the relative gene expression levels normalized by the standard deviation. Color legend is reported.

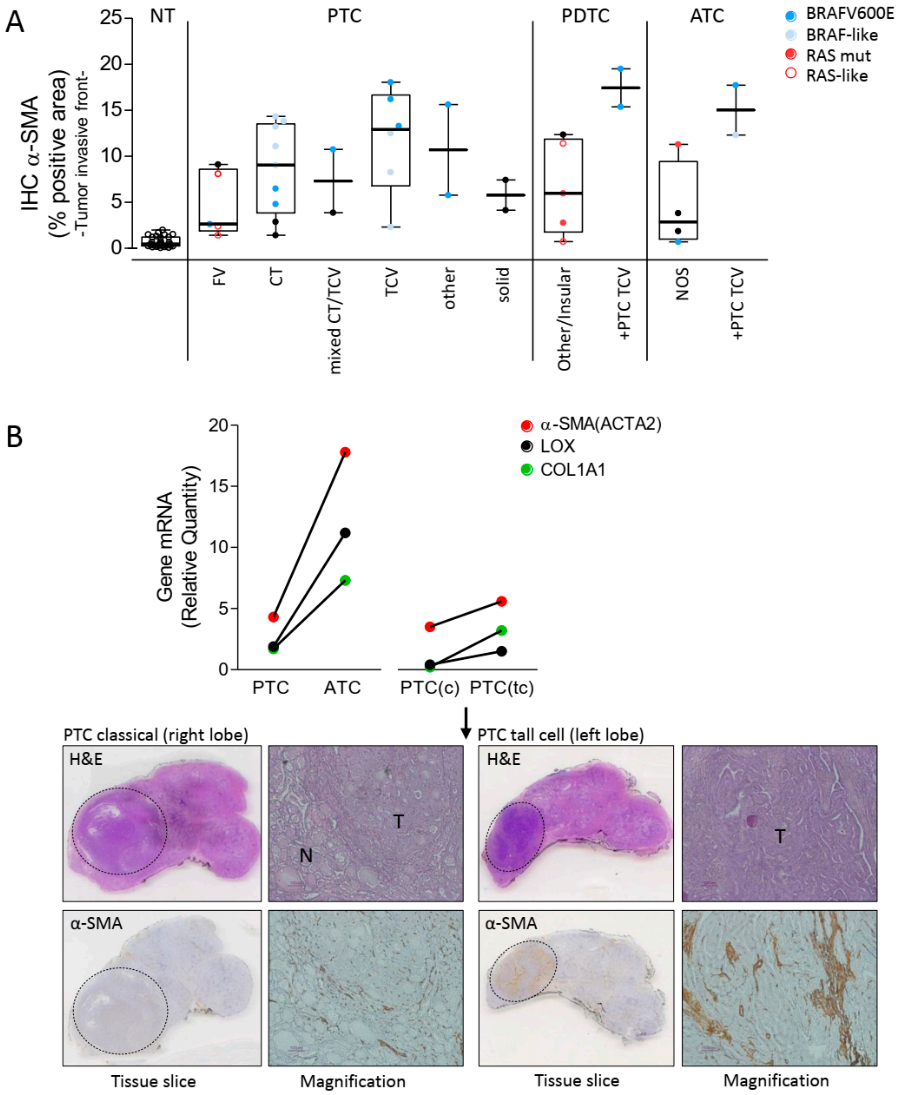

Figure S7. A. CAFs assessment by α-SMA IHC staining in human thyroid cancers stratified for tumor histotype, variant and gene drivers. Boxplot with scatterplot showing α-SMA IHC results by image digital quantification. Each dot represents the mean of 2-3 fields scored for samples; for tumor samples data about invasive front are specifically showed. Color legend indicates gene drivers or BRAF-/RAS-like signaling; tumors with unknown gene drivers or signaling are in black. **B.** *α-SMA*, *LOX* and *COL1A1* gene expression by quantitative RT-PCR in matched tumor tissues from 2 patients of our case list. Below representative H&E and α-SMA immunostained tissue sections and the corresponding magnification for the indicated patient. NT, non-neoplastic thyroid; PTC, papillary thyroid carcinoma; PDTC, poorly differentiated thyroid carcinoma; ATC, anaplastic thyroid carcinoma; FV, follicular variant; CT, classical variant; TCV, tall cell variant; NOS, not otherwise specified.

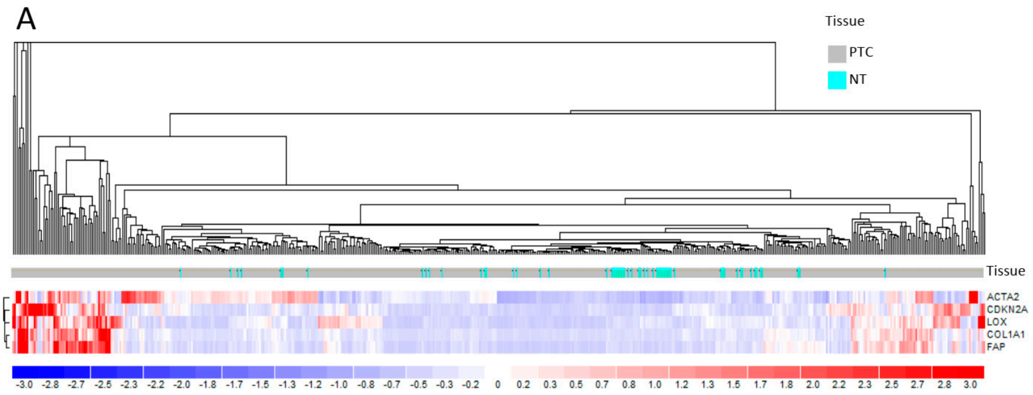

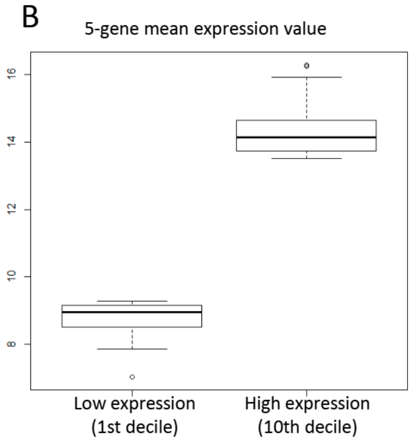

C

5-genes mean expression value	LN metastases number of patients (%)		
	N0	N1	
Low expression (1st decile)	34 (77%)	10 (23%)	p-value < 0.0001
High expression (10th decile)	9 (20%)	35 (80%)	

Figure S8. A. Unsupervised hierarchical clustering analysis of 486 papillary thyroid carcinomas (PTCs) and 58 non- neoplastic thyroids (NTs) from TGCA dataset on the five target genes expression. *α-SMA* and *p16* are indicated by the corresponding gene symbol, *ACTA2* and *CDKN2A*, respectively. The color scale bar represents the relative gene expression level normalized by the standard deviation. Color legend is reported for tissue type. **B**. Boxplot of the 5 gene mean expression value (log scale) calculated for 436 PTCs from TGCA for which clinical data about lymph node status (N0, N1, N1a, N1b) was available. The 1st decile and the 10th decile are specifically showed; Wilcoxon rank sum test measures the highly significant difference (p-value < 2.2×10^{-16}) between this two groups (44 samples in each group). **C.** Distribution of PTC patients stratified for the 5-gene mean expression class in relation to the absence (N0) or presence (N1/N1a/N1b) of lymph node (LN) metastases. The statistically significance by two-tailed Fisher's exact test is indicated.

Table S1. Human thyroid tumors clinicopathological features.

	ID	Histotype	Variant	Age	Gender	T	N	M	Specimen Preservation	Driving lesion	GEP	BRAF-/RAS-signaling	Paired RNA from NT
**A**	1	PTC	TCV	50	F	T2-3	N0	MX	FFPE	**BRAFV600E**	NA	NA	Yes
	2	PTC+PDTC	TCV	61	F	T3	N0	MX	FFPE	**BRAFV600E**	NA	NA	Yes
	3	PTC+PDTC	TCV	49	F	T3	N0	MX	FFPE	**BRAFV600E**	NA	NA	No
	4	PTC(+ATC)	TCV	85	F	NA	NA	NA	FFPE	**BRAFV600E**	NA	NA	No
	5	PTC	TCV	63	F	T2	N0	MX	FFPE	wt	NA	NA	No
	6	PTC	Classical	22	F	T4a	N1b	M1	FFPE	wt	NA	NA	No
	7	ATC	NOS	67	F	NA	NA	NA	FFPE	wt	NA	NA	No
	8	ATC+PTC	spindle(ATC);TCV(PTC)	70	M	NA	NA	NA	FFPE	**NRASQ61K**	NA	NA	No
	9	PTC	Classical + TCV	44	M	T4	N1a	MX	FFPE	**BRAFV600E**	NA	NA	Yes
	10	PTC	Classical	60	F	T2	N0	MX	FFPE	RET/PTC1	NA	NA	No
	11	PDTC+ATC	NOS	49	M	T4a	N1b	MX	FFPE	TRK fusion	NA	NA	No
	12	PTC	Classical+TCV	31	F	NA	NA	NA	FFPE	TRK fusion	NA	NA	Yes
	13	ATC	NOS	77	F	NA	NA	NA	FFPE	**BRAFV600E**	NA	NA	No
	14	ATC	NOS	73	F	NA	NA	NA	FFPE	wt	NA	NA	Yes
	15	PTC	Other	64	F	T3	N0	MX	FFPE	**BRAFV600E**	NA	NA	No
	16	PTC	Other	45	F	T4	N1a	MX	FFPE	**BRAFV600E**	NA	NA	No
	17	PTC	TCV	35	M	T3	N1b	MX	FFPE	**BRAFV600E**	NA	NA	No
	18	PTC	FV	42	M	T1	N0	MX	FFPE	wt	NA	NA	No
	19	PTC	FV	30	M	T1	N0	MX	FFPE	**BRAFV600E**	NA	NA	No
	20	PTC[1]	solid/trabecular	29	M	T4a	N1b	M1	FFPE	NA	NA	NA	No
	21	PDTC+ATC	TCV(PDTC);small cells (ATC)	67	F	T4a	N1b	M1	FFPE/Frozen	wt	Yes	**BRAF**-like	No
	22	PTC	Classical	26	F	T3	N1a	M0	FFPE/Frozen	RET fusion	Yes	**BRAF**-like	Yes
	23	PTC	Classical	46	M	T3	N1a	M0	FFPE/Frozen	wt	Yes	**BRAF**-like	No
	24	PDTC	Insular	73	M	T4a	N1b	M1	FFPE/Frozen	wt	Yes	**RAS**-like	No
	25	PTC	Classical	38	F	T3m	N1b	M0	FFPE/Frozen	wt	Yes	**BRAF**-like	No
	26	PTC	Classical	35	M	T3m	N1a	M0	FFPE/Frozen	wt	Yes [2]	**BRAF**-like	No
	27	PTC	FV	36	M	T3	NX	M0	FFPE/Frozen	wt	Yes	**RAS**-like	Yes
	28	PDTC	Insular	41	M	T3m	NX	M0	FFPE/Frozen	**HRASQ61K**	Yes	**BRAF**-like [3]	No
	29	PDTC+PTC	Insular(PDTC);TCV(PTC)	74	M	T3	NX	M1	FFPE/Frozen	**KRASQ61R**	Yes	**RAS**-like	No
	30	PTC	Solid	72	F	T3	N1b	M1	FFPE/Frozen	wt	Yes	**RAS**-like	No
	31	PTC	FV	51	F	T3	N0	M0	FFPE/Frozen	wt	Yes	**RAS**-like	Yes
	32	PTC	Classical	33	F	T3	N1b	M0	FFPE/Frozen	RET fusion	Yes	**BRAF**-like	No
	33	PTC	Classical	38	F	T3	NX	M0	FFPE/Frozen	**BRAFV600E**	Yes	**BRAF**-like	Yes
	34	PTC	TCV	48	F	T3	N1a	M0	FFPE/Frozen	wt	Yes	**BRAF**-like	Yes
	35	PDTC+PTC	Insular(PDTC);TCV(PTC)	52	F	T3m	NX	M0	FFPE/Frozen	wt	Yes	**RAS**-like	Yes

	36	PTC	Classical	37	F	T2	N1a	M0	FFPE/Frozen	**BRAFV600E**	Yes	**BRAF**-like	Yes
	37	PTC	TCV	75	F	T3m	N1a	M1	FFPE/Frozen	wt	Yes	**BRAF**-like	No
	38	PTC	TCV	70	F	T3m	N1b	M0	FFPE/Frozen	**BRAFV600E**	Yes	**BRAF**-like	No
	39	PDTC	mixed Insular/TCV	74	M	T3	NX	M1	FFPE/Frozen	**KRASQ61R**	Yes	**RAS**-like	No
	40	PTC	FV	38	F	2	NX	M0	FFPE/Frozen	wt	Yes	**RAS**-like	No
	41	PTC	TCV	51	F	T3	N0	M0	FFPE/Frozen	**BRAFV600E**	Yes	**BRAF**-like	Yes
	42	PTC	FV	52	F	T3	NX	M0	FFPE/Frozen	RET fusion	Yes	**RAS**-like	No
	43	PTC	FV	39	F	T3	NX	M0	FFPE/Frozen	**NRASQ61R**	Yes	**RAS**-like	No
**B**		PTC [1]	Classical	44	F	NA	NA	NA	Frozen	NA	Yes	**BRAF**-like	NA
		PTC [1]	TCV	35	F	T3m	N1b	M0	Frozen	**BRAFV600E**	Yes	**BRAF**-like	NA
		PTC+PDTC	FV(PTC)	44	F	T3	NX	M1	Frozen	NA	Yes	**RAS**-like	NA
		PTC	TCV	61	F	T2m	N1a	M0	Frozen	NA	Yes	**BRAF**-like	NA
		PTC	Classical	58	M	T1	N1b	M1	Frozen	NA	Yes	**RAS**-like	NA

Abbreviation: PTC, papillary thyroid carcinoma; PDTC, poorly differentiated thyroid carcinoma; ATC, anaplastic thyroid carcinoma; TCV, tall cell variant; FV, follicular variant; NOS, not otherwise specified GEP, gene expression profile; NA, not available; wt, wild type referred to the tested driving lesions (see materials and methods). [1] LFN metastasis; [2] Both Primary and a LFN metastasis were profiled by GEP; [3] Possible aggressive case with RAS mutation but BRAF-like signaling as described in reference [5]. **A**. The series of 43 TC patients investigated in the present study. B. Five additional TC patients for which only frozen tissue and gene expression profiles were available. This samples are included in the gene expression analysis reported in Figure 5.

Table S2. Driving lesion and BRAF-/RAS-signaling in the 254 tumor samples from GEO datasets.

	Type	Lesion	Signaling		Type	Lesion	Signaling		Type	Lesion	Signaling
1	PTC	**BRAF** mut	**BRAF-like**	**55**	PTC	RET fusion	**BRAF-like**	109	PTC	Unknown 2	**BRAF-like**
2	PTC	**BRAF** mut	**BRAF-like**	**56**	PTC	RET fusion	**BRAF-like**	110	PTC	Unknown 2	**BRAF-like**
3	PTC	**BRAF** mut	**BRAF-like**	**57**	PTC	RET fusion	**BRAF-like**	111	PTC	Unknown 2	**BRAF-like**
4	PTC	**BRAF** mut	**BRAF-like**	**58**	PTC	RET fusion	**BRAF-like**	112	PTC	Unknown 2	**BRAF-like**
5	PTC	**BRAF** mut	**BRAF-like**	**59**	PTC	RET fusion	**BRAF-like**	113	PTC	Unknown 3	**BRAF-like**
6	PTC	**BRAF** mut	**BRAF-like**	**60**	PTC	RET fusion	**BRAF-like**	114	PTC	Unknown 1	**RAS-like**
7	PTC	**BRAF** mut	**BRAF-like**	**61**	PTC	RET fusion	**BRAF-like**	115	PTC	Unknown 1	**RAS-like**
8	PTC	**BRAF** mut	**BRAF-like**	**62**	PTC	RET fusion	**BRAF-like**	116	PTC	Unknown 1	**RAS-like**
9	PTC	**BRAF** mut	**BRAF-like**	**63**	PTC	RET fusion	**BRAF-like**	117	PTC	Unknown 1	**RAS-like**
10	PTC	**BRAF** mut	**BRAF-like**	**64**	PTC	RET fusion	**BRAF-like**	118	PTC	Unknown 2	**RAS-like**
11	PTC	**BRAF** mut	**BRAF-like**	**65**	PTC	RET fusion	**BRAF-like**	119	PTC	Unknown 2	**RAS-like**
12	PTC	**BRAF** mut	**BRAF-like**	**66**	PTC	RET fusion	**BRAF-like**	120	PTC	Unknown 2	**RAS-like**
13	PTC	**BRAF** mut	**BRAF-like**	**67**	PTC	RET fusion	**BRAF-like**	121	PTC	Unknown 3	**RAS-like**
14	PTC	**BRAF** mut	**BRAF-like**	**68**	PTC	RET fusion	**BRAF-like**	122	PTC	Unknown 3	**RAS-like**
15	PTC	**BRAF** mut	**BRAF-like**	**69**	PTC	RET fusion	**BRAF-like**	123	PTC	Unknown 3	**RAS-like**
16	PTC	**BRAF** mut	**BRAF-like**	**70**	PTC	RET fusion	**BRAF-like**	124	PTC	Unknown 3	**RAS-like**
17	PTC	**BRAF** mut	**BRAF-like**	**71**	PTC	RET fusion	**RAS-like**	125	PTC	Unknown 2	**BRAF-like**
18	PTC	**BRAF** mut	**BRAF-like**	**72**	PTC	RET fusion	**RAS-like**	126	PTC	Unknown 2	**BRAF-like**
19	PTC	**BRAF** mut	**BRAF-like**	**73**	PTC	RET fusion	**RAS-like**	127	PTC	Unknown 2	**BRAF-like**
20	PTC	**BRAF** mut	**BRAF-like**	**74**	PTC	**RAS** mut	**RAS-like**	128	PTC	Unknown 4	**BRAF-like**
21	PTC	**BRAF** mut	**BRAF-like**	**75**	PTC	**RAS** mut	**RAS-like**	129	PTC	Unknown 4	**BRAF-like**
22	PTC	**BRAF** mut	**BRAF-like**	**76**	PTC	**RAS** mut	**RAS-like**	130	PTC	Unknown 4	**RAS-like**
23	PTC	**BRAF** mut	**BRAF-like**	**77**	PTC	**RAS** mut	**RAS-like**	131	PTC	Unknown 4	**BRAF-like**
24	PTC	**BRAF** mut	**BRAF-like**	**78**	PTC	**RAS** mut	**RAS-like**	132	PTC	Unknown 4	**BRAF-like**
25	PTC	**BRAF** mut	**BRAF-like**	**79**	PTC	**RAS** mut	**BRAF-like** **	133	PTC	Unknown 4	**RAS-like**
26	PTC	**BRAF** mut	**BRAF-like**	**80**	PTC	PAX8/PPARG	**RAS-like**	134	PTC	Unknown 4	**BRAF-like**
27	PTC	**BRAF** mut	**BRAF-like**	**81**	PTC	Unknown 1	**BRAF-like**	135	PTC	Unknown 4	**BRAF-like**
28	PTC	**BRAF** mut	**BRAF-like**	**82**	PTC	Unknown 1	**BRAF-like**	136	PTC	Unknown 4	**BRAF-like**
29	PTC	**BRAF** mut	**BRAF-like**	**83**	PTC	Unknown 1	**BRAF-like**	137	PDTC	**BRAF** mut	**BRAF-like**
30	PTC	**BRAF** mut	**BRAF-like**	**84**	PTC	Unknown 1	**BRAF-like**	138	PDTC	**BRAF** mut	**BRAF-like**
31	PTC	**BRAF** mut	**BRAF-like**	**85**	PTC	Unknown 1	**BRAF-like**	139	PDTC	RET fusion	**BRAF-like**
32	PTC	**BRAF** mut	**BRAF-like**	**86**	PTC	Unknown 1	**BRAF-like**	140	PDTC	ALK fusion	**RAS-like**
33	PTC	**BRAF** mut	**BRAF-like**	**87**	PTC	Unknown 1	**BRAF-like**	141	PDTC	**RAS** mut	**RAS-like**
34	PTC	**BRAF** mut	**BRAF-like**	**88**	PTC	Unknown 1	**BRAF-like**	142	PDTC	**RAS** mut	**RAS-like**
35	PTC	**BRAF** mut	**BRAF-like**	**89**	PTC	Unknown 1	**BRAF-like**	143	PDTC	**RAS** mut	**RAS-like**
36	PTC	**BRAF** mut	**BRAF-like**	**90**	PTC	Unknown 1	**BRAF-like**	144	PDTC	**RAS** mut	**RAS-like**
37	PTC	**BRAF** mut	**BRAF-like**	**91**	PTC	Unknown 1	**BRAF-like**	145	PDTC	**RAS** mut	**RAS-like**

38	PTC	**BRAF** mut	**BRAF-like**	**92**	PTC	Unknown 1	**BRAF-like**	146	PDTC	**RAS** mut	**RAS-like**
39	PTC	**BRAF** mut	**BRAF-like**	**93**	PTC	Unknown 1	**BRAF-like**	147	PDTC	**RAS** mut	**RAS-like**
40	PTC	**BRAF** mut	**BRAF-like**	**94**	PTC	Unknown 1	**BRAF-like**	148	PDTC	Unknown 5	**RAS-like**
41	PTC	**BRAF** mut	**BRAF-like**	**95**	PTC	Unknown 1	**BRAF-like**	149	PDTC	Unknown 5	**RAS-like**
42	PTC	**BRAF** mut	**BRAF-like**	**96**	PTC	Unknown 1	**BRAF-like**	150	PDTC	Unknown 5	**RAS-like**
43	PTC	**BRAF** mut	**BRAF-like**	**97**	PTC	Unknown 1	**BRAF-like**	151	PDTC	Unknown 5	**RAS-like**
44	PTC	**BRAF** mut	**BRAF-like**	**98**	PTC	Unknown 1	**BRAF-like**	152	PDTC	Unknown 5	**RAS-like**
45	PTC	**BRAF** mut	**BRAF-like**	**99**	PTC	Unknown 1	**BRAF-like**	153	PDTC	Unknown 5	**RAS-like**
46	PTC	**BRAF** mut	**RAS-like**	**100**	PTC	Unknown 1	**BRAF-like**	154	PDTC	**BRAF** mut	**BRAF-like**
47	PTC	RET fusion	**BRAF-like**	**101**	PTC	Unknown 1	**BRAF-like**	155	PDTC	Unknown 2	**BRAF-like**
48	PTC	RET fusion	**BRAF-like**	**102**	PTC	Unknown 1	**BRAF-like**	156	PDTC	Unknown 2	**BRAF-like**
49	PTC	RET fusion	**BRAF-like**	**103**	PTC	Unknown 1	**BRAF-like**	157	PDTC	Unknown 3	**BRAF-like**
50	PTC	RET fusion	**BRAF-like**	**104**	PTC	Unknown 1	**BRAF-like**	158	PDTC	Unknown 3	**RAS-like**
51	PTC	RET fusion	**BRAF-like**	**105**	PTC	Unknown 2	**BRAF-like**	159	FTC	**RAS** mut	**RAS-like**
52	PTC	RET fusion	**BRAF-like**	**106**	PTC	Unknown 2	**BRAF-like**	160	FTC	PAX8/PPARG	**RAS-like**
53	PTC	RET fusion	**BRAF-like**	**107**	PTC	Unknown 2	**BRAF-like**	161	FTC	Unknown 3	**RAS-like**
54	PTC	RET fusion	**BRAF-like**	**108**	PTC	Unknown 2	**BRAF-like**	162	FTC	Unknown 3	**RAS-like**

Table S2. *Cont.*

	Type	Lesion	Signaling		Type	Lesion	Signaling
163	ATC	**BRAF** mut	**BRAF-like**	214	ATC	NA	**BRAF-like**
164	ATC	**BRAF** mut	**BRAF-like**	215	PTC	NA	**BRAF-like**
165	ATC	**BRAF** mut	**BRAF-like**	216	PTC	NA	**BRAF-like**
166	ATC	**BRAF** mut	**BRAF-like**	217	PTC	NA	**BRAF-like**
167	ATC	**BRAF** mut	**BRAF-like**	218	PTC	NA	**BRAF-like**
168	ATC	**BRAF** mut	**BRAF-like**	219	PTC	NA	**RAS-like**
169	ATC	**BRAF** mut	**BRAF-like**	220	PTC	NA	**BRAF-like**
170	ATC	**BRAF** mut	**BRAF-like**	221	PTC	NA	**RAS-like**
171	ATC	**BRAF** mut	**BRAF-like**	222	PTC	NA	**BRAF-like**
172	ATC	**BRAF** mut	**BRAF-like**	223	PTC	NA	**BRAF-like**
173	ATC	**RAS** mut	**BRAF-like** *	224	PTC	NA	**BRAF-like**
174	ATC	**RAS** mut	**BRAF-like** *	225	PTC	NA	**BRAF-like**
175	ATC	Other	**BRAF-like**	226	PTC	NA	**RAS-like**
176	ATC	Unknown 5	**BRAF-like**	227	PTC	NA	**BRAF-like**
177	ATC	**RAS** mut	**BRAF-like** *	228	PTC	NA	**RAS-like**
178	ATC	**RAS** mut	**BRAF-like** *	229	PTC	NA	**BRAF-like**
179	ATC	**RAS** mut	**BRAF-like** *	230	PTC	NA	**BRAF-like**
180	ATC	Other	**BRAF-like**	231	PTC	NA	**BRAF-like**
181	ATC	**BRAF** mut	**RAS-like**	232	PTC	NA	**BRAF-like**
182	ATC	Other	**RAS-like**	233	PTC	NA	**BRAF-like**

183	ATC	NA	**BRAF-like**	234	PTC	NA	**BRAF-like**
184	ATC	NA	**BRAF-like**	235	PTC	NA	**BRAF-like**
185	ATC	NA	**BRAF-like**	236	PTC	NA	**RAS-like**
186	ATC	NA	**RAS-like**	237	PTC	NA	**BRAF-like**
187	ATC	NA	**BRAF-like**	238	PTC	NA	**BRAF-like**
188	ATC	NA	**BRAF-like**	239	PTC	NA	**BRAF-like**
189	ATC	NA	**RAS-like**	240	PTC	NA	**BRAF-like**
190	ATC	NA	**BRAF-like**	241	PTC	NA	**BRAF-like**
191	ATC	NA	**BRAF-like**	242	PTC	NA	**BRAF-like**
192	ATC	NA	**BRAF-like**	243	PTC	NA	**BRAF-like**
193	ATC	NA	**BRAF-like**	244	PTC	NA	**BRAF-like**
194	ATC	NA	**BRAF-like**	245	PTC	NA	**BRAF-like**
195	ATC	NA	**BRAF-like**	246	PTC	NA	**RAS-like**
196	ATC	NA	**BRAF-like**	247	PTC	NA	**BRAF-like**
197	ATC	NA	**BRAF-like**	248	PTC	NA	**BRAF-like**
198	ATC	NA	**BRAF-like**	249	PTC	NA	**BRAF-like**
199	ATC	NA	**BRAF-like**	250	PTC	NA	**BRAF-like**
200	ATC	NA	**BRAF-like**	251	PTC	NA	**BRAF-like**
201	ATC	NA	**BRAF-like**	252	PTC	NA	**BRAF-like**
202	ATC	NA	**BRAF-like**	253	PTC	NA	**BRAF-like**
203	ATC	NA	**BRAF-like**	254	PTC	NA	**BRAF-like**
204	ATC	NA	**BRAF-like**				
205	ATC	NA	**BRAF-like**				
206	ATC	NA	**BRAF-like**				
207	ATC	NA	**BRAF-like**				
208	ATC	NA	**BRAF-like**				
209	ATC	NA	**BRAF-like**				
210	ATC	NA	**BRAF-like**				
211	ATC	NA	**BRAF-like**				
212	ATC	NA	**BRAF-like**				
213	ATC	NA	**BRAF-like**				

NA: Not available; [1] BRAF mut and RET fusion wt; [2] BRAF mut wt; [3] RAS mut wt; [4] RET fusion wt; [5] None of MSK-IMPACT panel; * RAS-mutant ATCs with BRAF-like signaling already described in Reference [5]. ** possible aggressive tumor resembling RAS-mutant ATCs with BRAF-like signaling *

© 2020 by the authors. Submitted for possible open access publication under the terms and conditions of the Creative Commons Attribution (CC BY) license (http://creativecommons.org/licenses/by/4.0/).
